# Impact of *Plasmodium falciparum *infection on the frequency of moderate to severe anaemia in children below 10 years of age in Gabon

**DOI:** 10.1186/1475-2875-8-166

**Published:** 2009-07-20

**Authors:** Marielle K Bouyou-Akotet, Arnaud Dzeing-Ella, Eric Kendjo, Diane Etoughe, Edgard B Ngoungou, Timothy Planche, Jean Koko, Maryvonne Kombila

**Affiliations:** 1 Department of Parasitology, Mycology and Tropical Medicine, Faculty of Medicine, Université des Sciences de la Santé (USS), Libreville-Gabon. Malaria Clinical Research Unit, Centre Hospitalier de Libreville, Gabon; 2 Institute of Tropical Medicine, University of Tübingen, Germany; 3 Department of Infectious Diseases, St. George's Hospital Medical School, Cranmer Terrace, London, UK; 4 Department of Paediatrics, Faculty of Medicine, Université des Sciences de la Santé (USS), Libreville, Gabon

## Abstract

**Background:**

Improving the understanding of childhood malarial anaemia may help in the design of appropriate management strategies.

**Methods:**

A prospective observational study over a two-year period to assess the burden of anaemia and its relationship to *Plasmodium falciparum *infection and age was conducted in 8,195 febrile Gabonese children.

**Results:**

The proportion of children with anaemia was 83.6% (n = 6830), higher in children between the ages of six and 23 months. Those under three years old were more likely to develop moderate to severe anaemia (68%). The prevalence of malaria was 42.7% and *P. falciparum *infection was more frequent in children aged 36–47 months (54.5%). The proportion of anaemic children increased with parasite density (*p *< 0.01). Most of infected children were moderately to severely anaemic (69.5%, *p *< 0.01). Infants aged from one to 11 months had a higher risk of developing severe malarial anaemia. In children over six years of age, anaemia occurrence was high (>60%), but was unrelated to *P. falciparum *parasitaemia.

**Conclusion:**

Malaria is one of the main risk factors for childhood anaemia which represents a public health problem in Gabon. The risk of severe malarial anaemia increases up the age of three years. Efforts to improve strategies for controlling anaemia and malaria are needed.

## Background

Anaemia is a common cause of paediatric morbidity and mortality in sub-Saharan Africa [1,2]. Its complex aetiology involves interactions between multiple factors, which include malaria, helminthiasis, and infection with Human Immunodeficiency Virus, bacterial infections, nutritional deficiencies and haemoglobinopathies. Severe anaemia is one of the major complications of malaria in children [3,4]. The age distribution of severe malarial anaemia suggests that the acquisition of immunity has a positive effect on haemoglobin levels. In highly endemic areas, susceptibility to severe malarial anaemia increases during the age periods when parasite density and the frequency of uncomplicated episodes of malaria are highest [5].

Malaria, in turn, is a leading cause of anaemia in endemic regions. The use of insecticide-treated bed nets and of chemoprophylaxis has been shown to increase mean haemoglobin concentrations in children [6]. Estimates of the mortality of malaria-associated anaemia range from 190,000–974,000 annually in children less than five years of age [4]. Malarial anaemia, in some holoendemic areas of *Plasmodium falciparum *transmission, is the most common clinical manifestation of malaria in children aged between one and four years, with cerebral malaria only occurring in rare cases [3]. A high prevalence of severe anaemia in a population of children hospitalized for severe malaria was previously reported in Libreville [7].

This study was designed to examine the relationship between *P. falciparum *infection and anaemia in children living in Libreville, Gabon.

## Methods

### Study area

This cross-sectional study was conducted from January 2001 to December 2002 in Libreville, the capital city of Gabon, where the climate is humid and tropical. In this country, malaria transmission is perennial, predominantly caused by *P. falciparum*, with an entomologic inoculation rate of about 50 infective bites per person annually [8].

The population of Gabon is estimated at 1.34 million. Approximately one quarter of Gabon's population lives in Libreville, and about 40% are less than 15 years old. This study was conducted at the Malaria Clinical Research Unit (MCRU) at the Centre Hospitalier de Libreville (CHL), the largest hospital in Gabon.

### Patients

All children presenting with fever between January 2001 and December 2002 were examined by a clinician. After a detailed clinical examination, children whose parents or guardians had given informed consent and who met the following criteria were included in the study: age ranging from 0 to 119 months, fever or a history of fever within the 24 hours preceding the consultation or admission. Children infected with a *Plasmodium *species other than *P. falciparum *were excluded, as were those with another confirmed cause of anaemia (known haemoglobinopathy, known immunosuppression or severe malnutrition). This study was approved by the institutional review board of the Gabonese Ministry of Health.

### Procedures

On arrival at the MCRU, blood (two ml) was drawn into an EDTA tube for analysis.

#### Malaria diagnosis

*Plasmodium falciparum *parasitaemia was determined as follows: thick and thin blood films were stained with 20% Giemsa, and examined for malaria parasites by two trained microscopists following a standard, quality-controlled procedure. Parasitaemia was expressed as the number of asexual forms of *P. falciparum *per microlitre, by calculating the average parasitaemia per microscopic field of 10 μl of blood spread on a fixed area (1.8 cm^2^) [9]. This test was considered negative if there were no asexual forms of *P. falciparum *in 100 high-power fields. Patients were classified according to the presence or absence of malarial parasite and were grouped into six categories based on parasite density in the blood: <1,000 parasites (p)/μl, 1,000–9,999/μl, 10,000–99,999/μl, 100,000–249,999/μl and ≥ 250,000/μl (hyperparasitaemia).

#### Haematological measurements

Haemoglobin (Hb) measurements were performed using a Coulter counter (SKTS, Coulter Corporation). Children with Hb concentrations of less than 11 g/dl were considered anaemic. Anaemia was classified according to the WHO classification: severe (Hb<5 g/dl), moderate (5 < Hb < 8 g/dl) and mild (8 ≤ Hb < 11 g/dl).

### Data analysis

Demographic, clinical and laboratory data of patients were recorded on a standardized data entry form, and entered into Epi-info version 6.0 (February 9, 2005 CDC Atlanta) database. Data were analysed with Stata 9.2 (Stata Corporation, College Station, TX USA). Differences between groups were assessed using chi-squared or Fisher's exact tests for proportions, Student's t-test and analysis of variance (ANOVA) or Kruskal-Wallis test as appropriate. Spearman's test was used to assess the correlation between continuous variables. A *p*-value of less than 0.05 was considered significant.

Associations between variables were identified in univariate analysis and crude odds ratios (OR) with 95% confidence intervals (95%CI). Variables found to be significantly associated, and those with a *p-*value less than 0.20 were included in a logistic regression model and eliminated, one by one, in a backward procedure on the basis of the adjusted odds ratios (aOR) to develop a model with the strongest relationships between risk factors and outcome (anaemia).

## Results

### Baseline characteristics of patients

The MCRU screened 9,338 children, aged between 0 to 10 years old with fever or history of fever within 24 hours, from January 2001 to December 2002. 8,195 of these children met the inclusion criteria. 6,459 (78.8%) were less than 60 months old. The prevalence of anaemia for the whole population of children over the age of one month was 83.6% (n = 6,830). The prevalence of malarial infection was 42.7% (n = 3,502).

### Haemoglobin status

Anaemia was significantly associated with age: the proportion of anaemic children was over 84% for the one to 47 month old children; it then decreased in older children to a rate of 64% (*p *< 0.01) (Table 1). The highest rates of anaemia were in children aged between 12 and 23 months compared to other age groups (OR: 3.5, 95% CI:1.7–8.4, *p *< 0.01). The prevalence of mild anaemia did not vary significantly with age (Table 1). Children less than 36 months old were significantly more likely to develop moderate or severe anaemia (68%: n = 1,972) than older children (*p *< 0.01). Above this age, the proportion of children presenting to hospital without anaemia rose from 18.7% (48–59 months) to 35.3% (108–119 months) (Table 1).

**Table 1:** Haemoglobin levels in relation with age

Age (months)	N^a^	Mean Hb (SD^b^) g/dL	Anaemia prevalence^c ^n (%)	Severe anaemia n (%)	Moderate anaemia n (%)	Mild anaemia n (%)	Non anaemic n (%)
1–5	456	8.7 ± 2.4	387 (84.9)	39 (8.6)	97 (21.3)	251 (55.0)	69 (15.1)
6–11	1113	8.4 ± 2.5	957 (86.0)	135 (12.1)	285 (25.6)	537 (48.3)	156 (14.1)
12–23	1998	8.0 ± 2.5	1781 (89.1)	263 (13.2)	602 (30.1)	916 (45.8)	217 (10.9)
24–35	1378	8.4 ± 2.3	1212 (88.0)	116 (8.4)	435 (31.6)	661 (48.0)	166 (12.0)
36–47	878	8.5 ± 2.3	748 (85.2)	82 (9.4)	241 (27.4)	425 (48.4)	130 (14.8)
48–59	611	9.0 ± 2.4	496 (81.3)	47 (7.6)	144 (23.6)	306 (50.1)	114 (18.7)
60–71	441	9.0 ± 2.4	343 (77.8)	33 (7.5)	101 (22.9)	209 (47.4)	98 (22.2)
72–83	341	9.5 ± 2.2	249 (73.0)	11 (3.2)	66 (19.4)	172 (50.4)	93 (27.0)
84–95	295	9.7 ± 2.1	208 (70.5)	7 (2.4)	52 (17.6)	149 (50.5)	87 (28.8)
96–107	230	9.6 ± 2.2	171 (74.3)	8 (3.5)	38 (16.5)	125 (54.3)	59 (25.6)
108–119	428	9.7 ± 2.6	277 (64.7)	28 (6.5)	68 (15.9)	181 (42.3)	151 (35.3)
Total	8169	8.6 ± 2.5	6830 (83.6)	769 (9.4)	2129 (26.1)	3932 (48.1)	1339 (16.4)

^a^: Number of patients; ^b ^Standard deviation; ^c ^Prevalence in the whole population

The mean (SD) Hb concentration for the whole population was 8.6 ± 2.5 g/dl; mean Hb concentration increased significantly with age in older children, but never exceeded 10.0 g/dl. The lowest mean Hb concentration was observed in children between 12 and 23 months old (i.e. 8.0 ± 2.5 g/dl) (Table 1). The mean Hb level was 8.7 ± 2.4 g/dl in children under one month old. Seventy three percent (n = 19) of these children were anaemic (i.e. Hb < 14.0 g/dl), and only one had an Hb level less than 8 g/dl.

### *Plasmodium falciparum *infection

**Figure 1:**
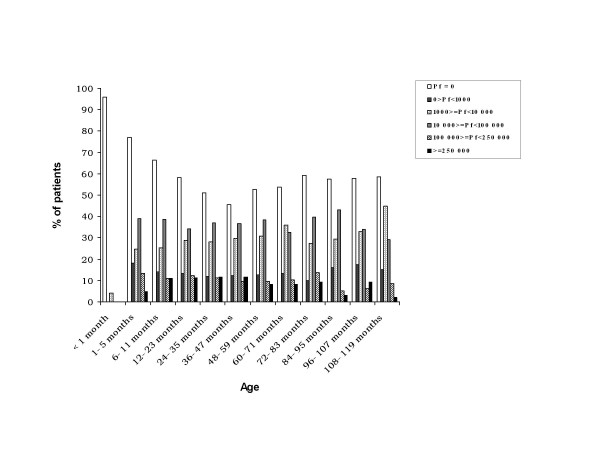
**Percentage of patients with different degrees of parasitaemia in relation with age groups**.

Of the 8,195 febrile children, 42.7% (3,502) had a positive blood film for malaria. The distribution of parasitaemia by age is shown in Figure 1. The frequency of *P. falciparum *infection increased from 23% in children aged one to five months to 54.6% for children between the ages of 36 and 47 months (*p *< 0.01). The prevalence of malaria remained constant at around 41% from the age of six years (Figure 1). Of the 3,502 children with *P. falciparum *infection, 106 (3%) were below six months of age, 2,653 (75.8%) were between six months and five years, and 743 (21.2%) were older than five years.

Hyperparasitaemia was observed in 11% (n = 270) of the children aged between six and 47 months, and rarely in children above the age of seven years (Figure 1). The geometric mean of parasite density was of 6,000/μl in children less than one month and increased until 15,730/μl in children between 24–35-months old; parasitaemia remained about 11,000 p/μl until 83 months, and subsequently decreased (*p *< 0.01).

### Relationship between *P. falciparum *infection and anaemia

**Figure 2:**
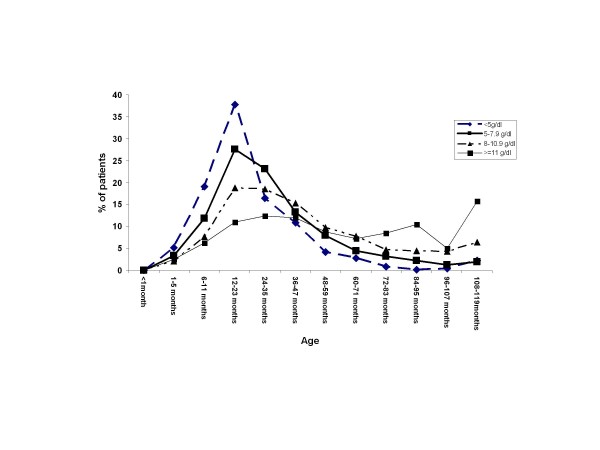
**Influence of age on anaemia occurring in parasitaemic children: distribution of patients according to anaemia and age**.

Anaemia was more common in *P. falciparum*-infected children (91%) than in uninfected children (77.7%) (*p *< 0.01). For children less than 83 months old, mean haemoglobin concentrations were significantly lower in malaria-infected patients than in uninfected patients (*p *< 0.01). Hb levels in children with *P. falciparum *infection varied significantly with age (6.9 g/dl to 9.7 g/dl, *p *< 0.01), but this relationship was not seen in children without *P. falciparum *infection (8.9 to 9.9 g/dl, *p*= 0.37) (Figure 2).

The geometric mean parasitaemia was significantly higher in the anaemic population (14,232/μl) than in the non-anaemic population (6,805/μl) (*p *< 0.01) and there was a negative correlation between parasitaemia and haemoglobin concentration (rho = -0.32, *p *< 0.01). The risk of anaemia was higher in hyperparasitaemic children OR:3.5 (95% CI 2.4–52; *p *< 0.01) (Table 2). The prevalence of anaemia among children also increased with the parasite count: 41.5% of children with a parasitaemia less than 1,000/μl had severe or moderate anaemia, 46% with 1,000 to <10,000/μl, 50% with 10,000 to <100,000/μL, 54.2% with parasitaemia between 100,000 and 249,000/μl and 66.7% with more than 250,000/μl had severe or moderate anaemia (*p *for χ^2 ^of trend <0.01). Mean log parasitaemia increased with age from one to 47 months and was significantly higher in children with severe anaemia from these age groups (*p *< 0.01).

**Table 2:** Multivariate analysis of risk factors for anaemia, malaria anaemia and severe malarial anaemia

	**Anaemia**			**Malarial anaemia**			**Severe malarial anaemia**		
			
	**aOR**	**[95% CI]**	***P***	**aOR**	**[95% CI]**	***P***	**aOR**	**[95% CI]**	***P***
**Age (months)**									
< 1	ND^*a*^			ND			ND		
1–5	1.00			2.55	[1.17–5.55]	0.014	8.55	[4.04–18.09]	<0.001
6–11	2.83	[1.22–6.56]	0.015	3.95	[2.41–6.49]	0.002	6.26	[4.12–9.49]	<0.001
12–23	3.45	[1.74–8.39]	0.001	4.27	[2.91–6.22]	<0.001	4.40	[3.26–5.88]	<0.001
24–35	3.15	[1.59–7.47]	0.001	4.04	[2.72–5.99]	<0.001	2.83	[1.86–4.33]	<0.001
36–47	2.16	[0.98–4.75]	0.056	3.63	[2.39–5.52]	<0.001	1.79	[1.10–2.89]	0.017
48–59	2.67	[1.20–5.95]	0.016	3.64	[2.22–5.80]	<0.001	0.89	[0.49–1.62]	NS
60–71	2.23	[0.98–5.07]	NS	2.64	[0.66–3.80]	NS	0.84	[0.41–1.73]	NS
72–83	2.21	[0.94–5.13]	NS	1.97	[0.65–3.30]	NS	0.83	[0.23–2.88]	NS
84–95	0.96	[0.36–2.58]	NS	1.29	[0.77–2.17]	NS	0.22	[0.03–1.87]	NS
96–107	0.80	[0.27–2.31]	NS	0.81	[0.30–2.67]	NS	0.44	[0.09–2.27]	NS
108–119	1.93	[0.81–4.57]	NS	1.13	[0.57–2.63]	NS	0.90	[0.41–1.98]	NS
									
**Parasitaemia**				ND^*b*^					
0	1.37	[1.11–2.28]	<0.001				1.00		
[0–1000]	1.75	[1.27–2.43]	<0.001				1.38	[0.92–2.08]	NS
[1000–10000]	2.20	[1.71–2.84]	<0.001				2.60	[2.07–3.26]	<0.001
[10000–100000]	2.04	[1.62–2.60]	<0.001				2.54	[2.07–3.13]	<0.001
[100000–250000]	2.70	[1.86–3.88]	<0.001				2.79	[2.03–3.83]	<0.001
≥ 250000	3.54	[2.43–5.17]	<0.001				4.96	[3.73–6.59]	<0.001

^a^: not significant ^b^: not determined

The risk of developing malarial anaemia was associated with age (OR = 2.92, 95% CI: 2.6–3.4, *p *< 0.01). It increased between the ages of one and 23 months, and decreased thereafter (*p *< 0.01) (Table 2). The proportion of parasitaemic children was greater in case of severe anaemia respectively of 1,403/3,892 (36%) children with mild anaemia, 1,193/2,087 (57.2%) of children with moderate anaemia, and 485/751 (64.6%) of children with severe anaemia (*p *< 0.01).

Overall, 84.3% (424/503) of children with severe malarial anaemia were six- to 47-months old. In the infected four to 10-year old-children, the prevalence of moderate to severe anaemia decreased significantly with age (*p *< 0.01) (Figure 2). The risk of developing severe malarial anaemia was higher in infants, increasing significantly with age until 24 months old, and then decreased until the age of 47 months; this risk had disappeared by the age of five years (Table 2).

## Discussion

Improving the understanding of childhood malarial anaemia may help in the design of appropriate management strategies. Data from 8,195 febrile children up to ten years old were analyzed. A high prevalence of anaemia (83.6%) was found in Gabonese children. This prevalence is comparable to that observed elsewhere in Africa [2,10-12]. One striking observation was the marked dependence of the prevalence of anaemia on age as reported from other malaria endemic areas [2,10,11]. In Libreville, the highest prevalence rate of anaemia (89.1%) occurred among the 12 to 35 months old children; this group also had the lowest mean Hb concentration. Nevertheless, anaemia was still frequent in older children (more than 60%), compared to that reported for similarly aged children elsewhere [2,11,12]. Moreover, the mean Hb concentration, which was generally low across the whole population (8.6 g/dl), increased with age from four years, but did not reach normal levels. Moderate to severe anaemia occurred more frequently in the first three years of life, as described by others [2,13,14]. All these observations highlight the burden of anaemia in Gabon.

The prevalence of microscopic *P. falciparum *infection was 42.3%, higher than that reported for children from another hospital in Libreville and in urban areas from Ghana and Zaire, but indistinguishable from that noticed in Cameroon and Togo [2,12,15-17].

The prevalence of malaria was also age-related, increasing from birth to 47 months, with children between 24 and 59 months having the highest risk of infection. More than 40% of febrile children over the age of five years were parasitaemic, this is not in agreement with the epidemiology of malaria in a hyperendemic region. Libreville is an urban area in which a shanty-town with high malaria transmission and a residential area with low transmission coexist. Urbanization impedes malaria transmission and would increase the number of non-immune individuals, possibly delaying premunition in non-immune individuals [18].

Malaria infection during the first few months of life is rare. In children living in a highly endemic area of western Kenya the mean time between birth and detectable parasitaemia was 3.4 months [5]. One of the 26 febrile children less than one month old was *P. falciparum*-infected. In Lambaréné, a city located at 263 km from Libreville, among 896 children aged up to three months, only one who was less than one month old had asymptomatic parasitaemia; none of these children had clinical malaria [19]. Symptomatic children were included at MCRU, as more than 95% of fever cases in babies were not attributable to malaria and, taking into account the findings of Lambaréné team, it appears that malaria is less frequent in children under the age of one year in Gabon.

The role of malaria in childhood anaemia is highlighted by the following factors: i) mean haemoglobin concentration was significantly lower in children with *P. falciparum *infection (7.8 g/dl) than in uninfected children (9.2 g/dl), ii) there was an increase in the proportion of anaemic children with increasing parasitaemia, and iii) geometric mean parasite density was significantly higher in the anaemic population (14232/μl) than in the non-anaemic population (6805/μl). Children with any degree of parasitaemia were at risk to develop anaemia. In addition to these factors identified, very low-density parasitaemia (common in areas of intense malaria transmission and reflecting either chronic low-grade infection or the tail-end of an acute high-density infection) has a significant impact on anaemia [2,20].

The magnitude of the impact of *P. falciparum *infection on anaemia was age-related and more pronounced in children between six months and five years of age, as previously reported [10,11,21,22]. Other factors may contribute to the occurrence of anaemia in children of six years and older. Asymptomatic parasitaemia, which accounts for 7% to 23% of all *P. falciparum *detected by microscopy, is also an independent factor influencing Hb levels [21,23,24].

The prevalence of mild anaemia did not vary with infection and was not age-related; on the other hand, moderate to severe malarial anaemia were strongly associated with parasitaemia in children less than four years old. Several studies performed in intense malaria transmission areas report a high rate of severe malarial anaemia in children less than three years old [2,7,11,13,25,26]. However, few of them found a correlation between parasite density and severe anaemia in young children. Severe malarial anaemia is age-related and the low prevalence of severe malarial anaemia in older children and adults may result from the capacity to control parasitaemia.

This study was not designed to assess all the main factors that could be associated with malarial anaemia. Other factors, unrelated to *P. falciparum *infection, may augment the effect of malaria, and predispose young children to a greater rate of anaemia during and after malarial infection. Red blood cell polymorphisms are causes of anaemia. In Gabon, the prevalence of sickle cell trait is 21 to 24% among children up to 15 years old, 1 to 3% of them are homozygous [27,28]. α-thalassemia is rare (0.3%) [28]. Both phenotypes have not been associated with significant variations of haematological parameters during malaria [28,29]. Socio-economic factors were not found to influence severe malarial anaemia, hyperparasitaemia or re-infection [30]. In addition, most of young children sleep under bed nets in the country. Helminth infections are frequent, but, self-medication with anti-helminthic medications of febrile children suffering from abdominal pain (a frequent symptom of malaria) is very common; moreover, these infections have not always been associated with malarial anaemia.

## Conclusion

The results of this study provide good baseline data on the burden of anaemia in Gabonese young children. *Plasmodium falciparum *infection plays an important role and remains unquestionably one of the main risk factors for childhood anaemia in Gabon. Children aged less than three years old have the greatest risk of severe malarial anaemia. Further investigation within the communities and longitudinal studies are needed to determine the role of host and parasite factors in the development and outcome of anaemia and malarial anaemia, and may aid in designing appropriate management strategies for the control of these significant public health problems.

## Competing interests

The authors declare that they have no competing interests.

## Authors' contributions

The study was divided and the article drafted by MK B-A, A D-E, and M K. It was conducted by A D-E and M K with contribution from all authors. Data were analysed by E K, EB N and MK B-A. MK B-A is the guarantor.

## Ethical Approval

Ministry of Public Health and Population, Gabon.
